# Supplementary material for: A Novel Score to Predict One-Year Mortality after Transcatheter Aortic Valve Replacement, Naples Prognostic Score
Source: Medicina (Kaunas). 2023 Sep 15;59(9):1666. doi: 10.3390/medicina59091666 (PMC10534754; doi:10.3390/medicina59091666)
Supplement: Supplementary file 1 [file medicina-59-01666-s001.zip › medicina-2532894-supplementary.pdf]

Supplement: Table S1. Univariate Analysis Appendices

Variable	p	EXP	95% C.I.for EXP	
			Lower	Upper
Age	0.332	1.027	0.973	1.084
Euroscore	<0.001	1.056	1.026	1.086
LvEF	0.983	1.000	0.972	1.028
Hemoglobin	0.028	0.780	0.626	0.974
Platelet count	0.226	1.000	1.000	1.000
Neutrophil	0.110	1.000	1.000	1.000
Lymphocyte	0.317	1.000	0.999	1.000
Monocyte	0.045	1.002	1.000	1.000
Creatinine	0.134	1.809	0.883	3.928
Total Cholesterol	0.530	1.003	0.995	1.011
LDL	0.103	1.008	0.998	1.017
HDL	0.090	0.971	0.939	1.005
Triglycerid	0.838	1.001	0.995	1.006
Total protein	0.006	0.942	0.903	0.983
Albumin	<0.001	0.887	0.821	0.938
NLR	0.090	1.078	0.988	1.175
LMR	0.008	0.690	0.525	0.906
Gender	0.102	1.841	0.886	3.824
DM	0.773	1.112	0.539	2.297
HT	0.371	1.957	0.450	8.507
AF	0.022	2.347	1.130	4.874
SVO	0.383	1.646	0.537	5.038
COPD	0.062	2.037	0.965	4.299
PCI	0.370	0.693	0.311	1.545
CABG	0.293	1.576	0.675	3.681
Balon exp	0.961	1.019	0.475	2.190
Naples group	0.008	2.859	1.313	6.222
